# Phase Resetting of the Mammalian Circadian Clock Relies on a Rapid Shift of a Small Population of Pacemaker Neurons

**DOI:** 10.1371/journal.pone.0025437

**Published:** 2011-09-22

**Authors:** Jos H. T. Rohling, Henk Tjebbe vanderLeest, Stephan Michel, Mariska J. Vansteensel, Johanna H. Meijer

**Affiliations:** 1 Laboratory for Neurophysiology, Department of Molecular Cell Biology, Leiden University Medical Center, Leiden, The Netherlands; 2 Section of Brain Function and Plasticity, Department of Neurology and Neurosurgery, Rudolf Magnus Institute of Neuroscience, University Medical Center Utrecht, Utrecht, The Netherlands; Vanderbilt University, United States of America

## Abstract

The circadian pacemaker of the suprachiasmatic nuclei (SCN) contains a major pacemaker for 24 h rhythms that is synchronized to the external light-dark cycle. In response to a shift in the external cycle, neurons of the SCN resynchronize with different pace. We performed electrical activity recordings of the SCN of rats in vitro following a 6 hour delay of the light-dark cycle and observed a bimodal electrical activity pattern with a shifted and an unshifted component. The shifted component was relatively narrow as compared to the unshifted component (2.2 h and 5.7 h, respectively). Curve fitting and simulations predicted that less than 30% of the neurons contribute to the shifted component and that their phase distribution is small. This prediction was confirmed by electrophysiological recordings of neuronal subpopulations. Only 25% of the neurons exhibited an immediate shift in the phase of the electrical activity rhythms, and the phases of the shifted subpopulations appeared significantly more synchronized as compared to the phases of the unshifted subpopulations (p<0.05). We also performed electrical activity recordings of the SCN following a 9 hour advance of the light-dark cycle. The phase advances induced a large desynchrony among the neurons, but consistent with the delays, only 19% of the neurons peaked at the mid of the new light phase. The data suggest that resetting of the central circadian pacemaker to both delays and advances is brought about by an initial shift of a relatively small group of neurons that becomes highly synchronized following a shift in the external cycle. The high degree of synchronization of the shifted neurons may add to the ability of this group to reset the pacemaker. The large desynchronization observed following advances may contribute to the relative difficulty of the circadian system to respond to advanced light cycles.

## Introduction

In mammals, the suprachiasmatic nuclei (SCN) of the anterior hypothalamus drive daily rhythms in physiological processes and behavior. Individual neurons of the SCN generate endogenous circadian rhythms in gene expression by means of transcriptional and translational feedback loops, resulting in autonomous oscillations at the single cell level [1]–[3]. Synchronization among SCN neurons results in a coherent rhythm at the tissue level of molecular and electrical activity [4], [5]. To gain adaptive significance, the SCN clock is synchronized to the external 24 hour environmental cycle [6], with light being the most important external signal. By stimulation of melanopsin containing ganglion cells, light information reaches the SCN through the retinohypothalamic tract, which innervates the ventral part of the rat SCN [6]–[8].

When exposed to changes in the external light-dark cycle, the circadian system requires several days to readjust. During these days, a temporal disruption of day to day rhythms is observed, which can be experienced as jet lag. The symptoms associated with jet lag are fatigue, reduced alertness and concentration, fragmented sleep, premature awakening, excessive sleepiness, and a decrement in performance [9], [10]. Previous studies have shown that the molecular and electrical rhythms of the SCN require several days to readjust to a shifted light-dark regime [11]–[15]. Molecular studies have shown an internal desynchronization within the SCN, caused by rapid resetting of the ventral part and slow resetting of the dorsal part of the SCN [13], [16]–[18]. For instance, the transcriptional rhythms of the period genes Per1 and Per2 shift rapidly in the ventrolateral SCN, but slower in the dorsomedial SCN [13]. Electrical activity measurements have revealed bimodal electrical activity patterns in the rat SCN following exposure to a shifted cycle [12]. One component of the bimodal pattern is fully reset to the new phase and corresponds with activity in the ventral SCN, while the other component is not significantly shifted and corresponds with activity in the dorsal SCN [12], [16]–[18]. While these insights were largely qualitative, the aim of the present study is to understand resetting kinetics of the SCN clock at the level of neuronal subpopulation activity, and to provide a quantitative model that can account for the adjustment of the SCN clock.

We first measured the pattern of electrical activity rhythm in SCN slices following a delaying shift of the light-dark cycle and observed bimodal patterns with one shifted component and another component that was not significantly shifted. Analysis of the bimodal activity records showed that the peak of the shifted component was narrow and the peak of the unshifted component broader. Recordings of neuronal subpopulations revealed that the neuronal activity patterns within the shifted component were highly synchronized in phase. We propose that phase shifts are brought about by an initial rapid shift of a relatively small subpopulation of SCN neurons that exhibits strong phase synchronization after exposure to a shift in the environmental cycle. Finally we tested our model suggesting the role of a small neuronal subpopulation in phase-resetting for the case of phase advances. We confirmed that a similar mechanism may underlie phase advancing responses of the SCN, but that a larger degree of desynchrony is observed within the SCN.

## Results

**Figure 1:**
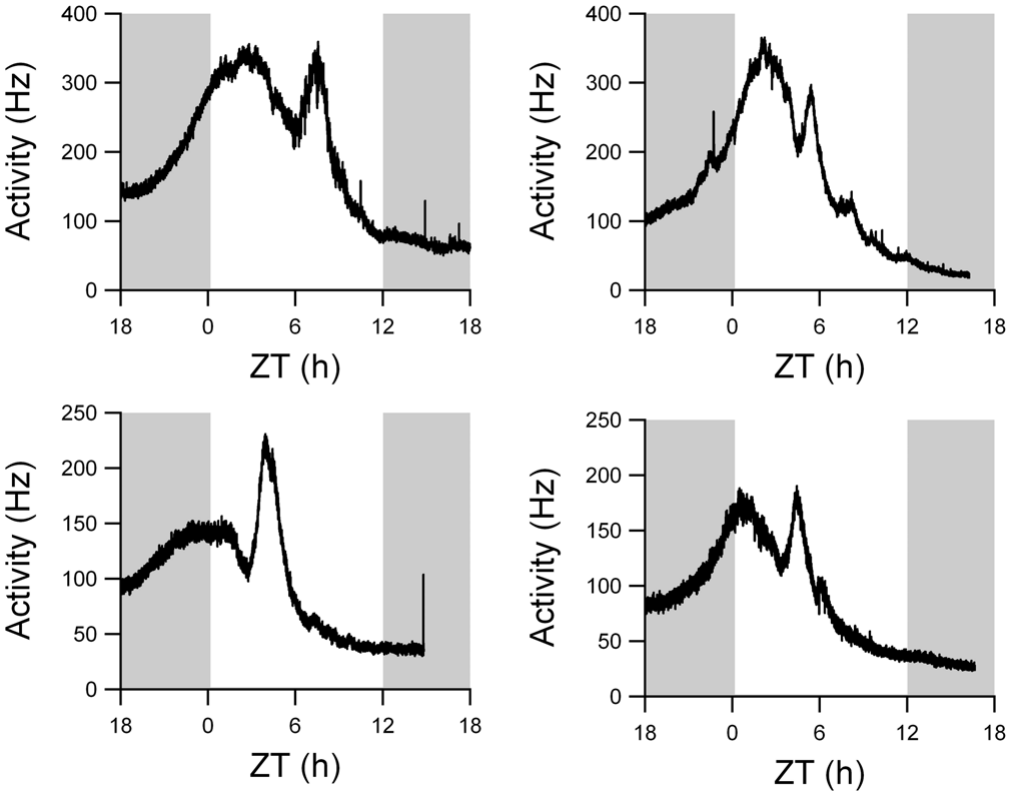
Four examples of bimodal multiunit electrical activity recordings, showing two characteristic components. The left peak in each recording is unshifted component, while the right peak is the shifted component. The shifted light-dark schedule is depicted in the background, with gray indicating night and white indicating day. Time axis depicts the new phase, following the shift.

**Figure 2:**
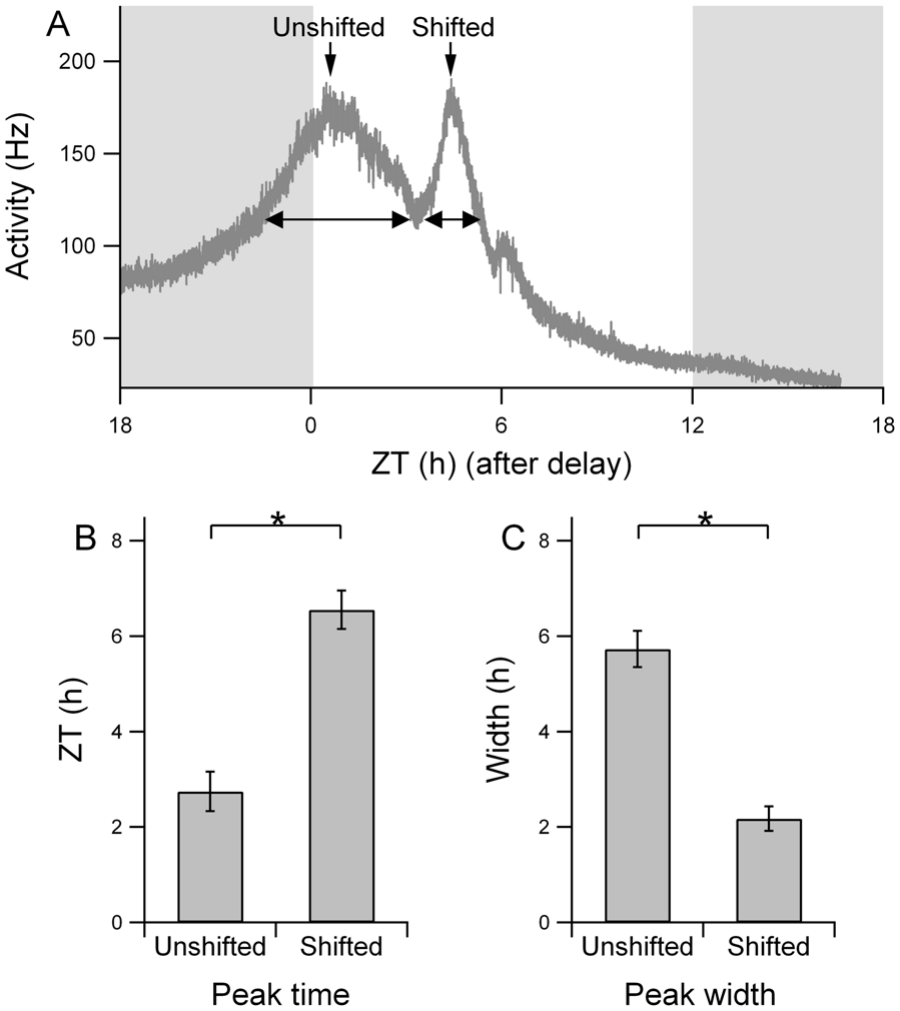
Width and time of peak activity for both components. (A) Raw multiunit activity data following a shift of the light-dark cycle. After smoothing of the data, the trough between both components was determined as well as the peak times. The width of the unshifted and the shifted component was determined by drawing a horizontal line from the trough to the opposing slope of the component's peak. The ZT refers to the shifted Zeitgeber time. (B) The peak time for the unshifted and shifted component was at ZT 2.7±0.4 h, and at ZT 6.6±0.4 h respectively. The difference in peak time was significant (p<0.01). (C) The width of the peak of the unshifted and shifted component was 5.7±0.4 h and 2.2±0.3 h, respectively, which was significantly different (p<0.01).

Rats were entrained to a 12:12 h light-dark cycle and then exposed to an abrupt 6 hour phase delay of the light-dark cycle. One day after the shift, electrical activity patterns were recorded in acutely prepared brain slices. In 41% of the recordings a bimodal pattern was obtained. We pooled these data with previously obtained data to allow for a quantitative analysis of the electrical activity pattern, rendering a total of 21 bimodal activity patterns (10 previous recordings [12] and 11 new recordings; examples in Figure 1). The peak times of the two components were significantly different (p<0.01, shifted component: Δϕ  =  −6.6±0.4 h, unshifted component: Δϕ  =  −2.7±0.4 h). It was evident from all recordings that the peak of the shifted component was narrower than the peak of the unshifted component (Figure 1). At the level of the trough between both peaks, the width of the shifted component was 2.2±0.3 h and was significantly smaller than the width of the unshifted component (5.7±0.4 h, p<0.001; Figure 2).

**Figure 3:**
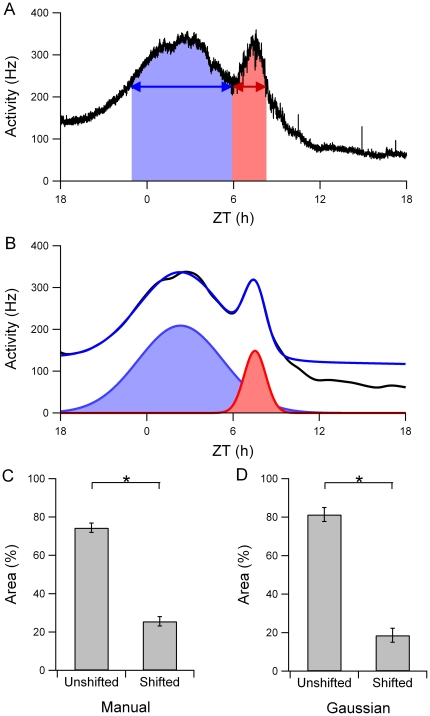
Relative number of action potentials contributing to each component. (A) The total area under each of the components was used to determine the relative number of action potentials contributing to each component. (B) Gaussian functions fitted to the smoothed multiunit activity pattern. The black line is the smoothed multiunit activity pattern. The blue line is the total fitted pattern, and the Gaussian functions are shown below the fitted signal. (C) The area of the unshifted component as determined in (A) was 74% of the total area, while the area of the shifted component was 26%. (D) The relative area of the unshifted component, as determined in (B), was 79%, and of the shifted component was 21%.

To determine the area under the curve for both components, we calculated the relative contribution of each component to the total surface using a manual curve fitting method (Figure 3A). The results showed that the area of the unshifted component was 74% and the area of the shifted component 26% of the total area (Figure 3C). We also fitted Gaussian functions to the smoothed pattern using the automated fitting procedures and determined the relative areas for the two components (Figure 3B). The unshifted component represented 79% and the shifted component 21% of the total electrical activity (Figure 3D).

### Simulations

**Figure 4:**
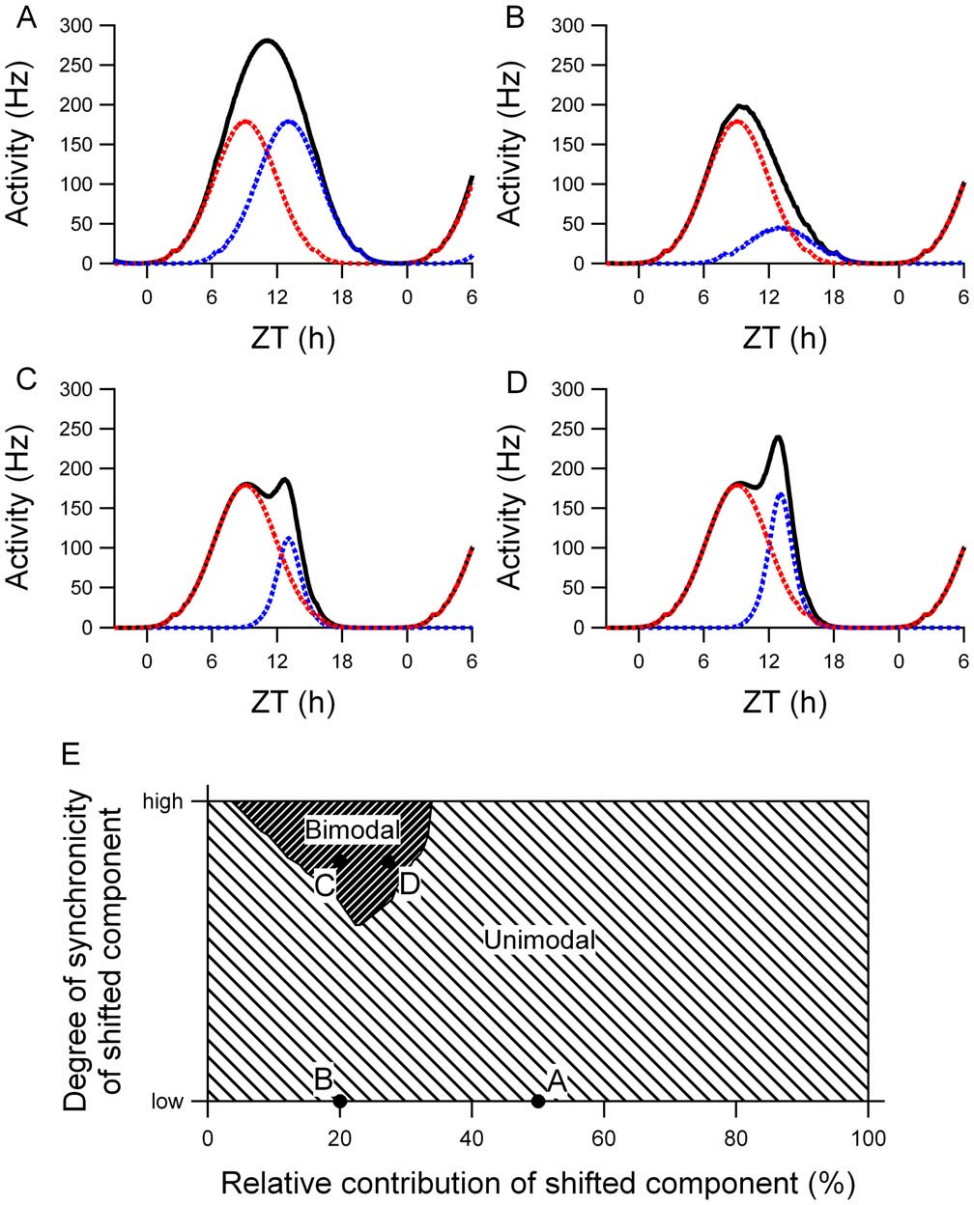
Simulations of electrical activity patterns with two components. The black line shows the resulting multiunit pattern of two components with peak activity at ZT 9 (red dotted line) and ZT 13 (blue dotted line). The neurons of each component show a Gaussian distribution with certain distribution-width (σ). (A) Both populations have the same number of neurons and the same distribution. This leads to a single peak in the multiunit activity pattern. (B) The number of neurons in the shifted component is decreased, resulting in a unimodal multiunit pattern. (C) The number of neurons in the shifted component is decreased and the distribution of the shifted component is narrower, which leads to a bimodal multiunit pattern. (D) If the number of neurons is slightly increased with respect to (C), and the distribution of the shifted component is still narrow, the second peak becomes rapidly higher, see also figure 1 C. (E) Search-space that shows the effect of the number of neurons in each component and of their relative distribution to the multiunit pattern. The multiunit pattern becomes bimodal when the number of neurons of the shifted component is relatively small as compared to the total number of neurons (x-axis) and when the degree of synchronization of the shifted component is high (y-axis). The examples shown in (A)-(D) are depicted by the dots in the search-space.

The two components of the rhythm in electrical activity were simulated by distributing two subpopulations of single unit patterns around ZT 9 and ZT 13. When both components were composed of an equal number of neurons with the same distribution, the resulting multiunit pattern was unimodal (Figure 4A). If the number of neurons in the shifted component was reduced to 20% of the total number of neurons, while the distribution was unchanged, the multiunit pattern was also unimodal (Figure 4B). A bimodal multiunit pattern was obtained when not only the number of neurons was reduced, but when also the distribution of the neurons in the shifted component was made considerably narrower compared to the distribution in the unshifted component (Figure 4C). Analytical testing of the parameter space resulted in the observation that in the presence of two underlying populations of neurons, bimodal peaks only occurred in a relatively small, restricted part of the parameter space (Figure 4E). This means that the occurrence of bimodal peaks is ‘rare’ and only occurs when specific conditions are met. Unimodal peaks are more likely to be obtained, even if two underlying groups of oscillators are present.

Minor variations in the number of neurons in the shifted component readily produced differences in the height of the simulated shifted peak (Figure 4C, D). This can be understood as the neurons are highly synchronized in phase. As a consequence, small differences in neuron number lead immediately to differences in peak height. These differences in peak height were indeed observed in the experimental data (Figure 1). If the number of neurons contributing to the shifted component in the simulations was about 20% of the total number of neurons, a bimodal shape in the multiunit activity pattern was found with a similar peak height of the shifted and unshifted component, in accordance with our experimental findings (Figure 4C). If a slightly higher percentage of neurons contributed to the shifted component, the height of the shifted peak increased substantially as opposed to the height of the unshifted peak (Figure 4D).

### Subpopulation Analysis

**Figure 5:**
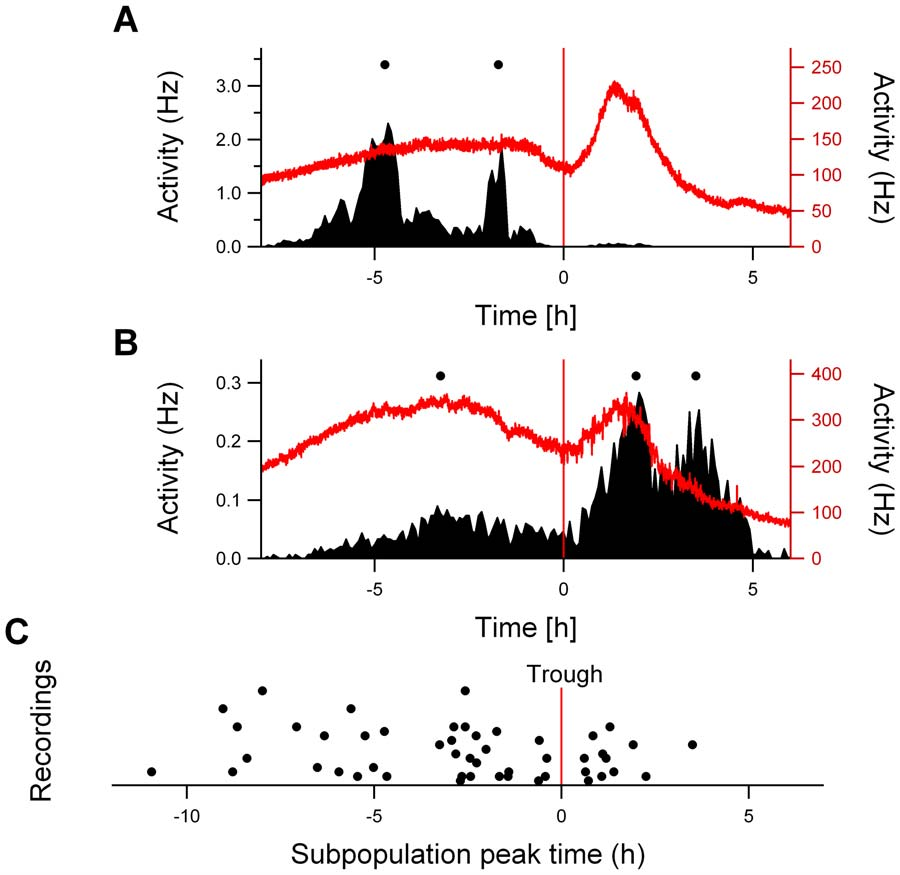
Subpopulation analysis of the electrical activity profile after the shift in LD. (A and B) Two examples of the subpopulations (left ordinate axis) and multiunit activity pattern (right ordinate axis) for the same recording. The peak times of the subpopulations, indicated by the dots, were determined relative to the trough of the multiunit pattern (indicated by the red vertical line). (C) The number of subpopulations found in the unshifted component was higher than that in the shifted component, and showed a broader distribution.

We isolated the subpopulations from the multiunit electrical activity data [see 20] to estimate the number of subpopulations in the unshifted and shifted component and to determine their phase distribution. The peak times of the subpopulations were assessed relative to the time of the trough in the population recording. The subpopulation analysis showed that more neurons were active before the trough than after the trough (Figure 5). Before the trough, a total number of 37 subpopulations showed their peak activity compared to 12 subpopulations after the trough. The distribution of the phases of subpopulation peaks after the trough was significantly narrower than before the trough (p<0.05).

### Phase Advances

**Figure 6:**
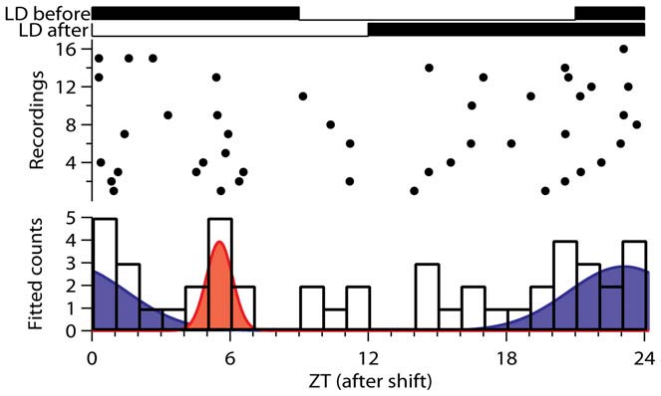
Subpopulation analysis of the electrical activity profile after an advancing shift. The peak times of the subpopulations, indicated by the dots, were determined relative to the light-dark cycle after an advancing shift of 9 h. The top lines of the figure show the LD cycles before the shift and after the shift of the light-dark cycle. In the lower figure the numbers of subpopulations per hour were fitted by two Gaussian functions. The proportion of subpopulations that shifted to the middle of the new light period was 19% of the total amount of subpopulations.

In addition to 6-h delay experiments we also subjected rats to a phase advance protocol. Previous studies showed that phase advances of 6 h did not result in a bimodal multiunit pattern [15], [19], which made us decide to subject the animals to an advancing shift of 9 hours. Out of 16 successful recordings, most recordings showed a broad peak, and the mean peak width was 14.2 h ± 5 h (n = 11). However, between the recorded patterns large variation was observed and only 2 recordings showed a bimodal peak. Subpopulation recordings showed a broad distribution of subpopulation activities over the 24-h period. From a total amount of 47 subpopulations, 9 subpopulations (19%) showed their maximal activity between ZT 4-7, while the other populations were broadly distributed (Figure 6).

## Discussion

We explored the phase shifting behavior of neuronal subpopulations in the SCN after an abrupt phase delay of the light-dark cycle. On the first day after the manipulation, a shifted and an unshifted component could be identified in the bimodal electrical activity pattern. An analysis at the level of the trough of the bimodal pattern revealed that the unshifted component was nearly three times as broad as the shifted component. We next calculated the relative contribution of each component to the ensemble pattern by the use of curve fitting methods. We found by manual fitting that the area representing the shifted component was 26% of the total area, while the unshifted component was 74%. In the mathematical curve fitting analysis, the shifted component was 21% of the total area and the unshifted component was 79%. The analysis, together with computer simulations lead to the predictions that (1) the number of neurons in the shifted component is a small fraction of the total number of neurons, and (2) the phase-synchrony between the shifted neurons is high, as compared to the phase-synchrony of the unshifted neuronal population.

Neuronal subpopulation recordings were performed to test these predictions and the peak times of the subpopulations were plotted relative to the trough in the multiunit recording. The analysis of subpopulation data showed that 25% of the neuronal subpopulations clustered at the mid of the new light cycle on the first day after a delay. The subpopulations showed a remarkable narrow phase distribution, as opposed to the unshifted subpopulations. The low percentage of shifted neurons as well as their high degree of synchronization were in agreement with the predictions from the simulations.

While 41% of our multiunit recordings showed bimodal patterns in electrical activity after exposure to the shifted light cycle, 59% of the multiunit recordings showed unimodal patterns, raising the question whether the unimodal patterns reflect different underlying resetting kinetics. We used simulations to investigate how the activity patterns of two subpopulations can lead to either a unimodal or bimodal ensemble pattern. In our simulations it became clear that a 4 h time difference between the two components, as observed in our data, may not be large enough to yield a bimodal activity pattern, but can also result in a unimodal activity pattern. Bimodal patterns in multiunit activity only arise when the number of neurons contributing to the shifted population is small compared to the unshifted population and when the phase distribution between the neurons of the shifted population is narrow compared to the phase distribution of the unshifted neurons. We expect therefore that the unimodal patterns do not reflect different resetting kinetics, but reflect the presence of two underlying components as well (see Figure 4).

In this study we were particularly interested in the phase distribution of neuronal subpopulations following a shifted cycle. If a neuronal population is highly synchronized in phase, coupled limit-cycle oscillator theory predicts that this population exerts a stronger phase shifting response on other, more randomly distributed neurons, than vice versa [20], [21]. This can be understood as the synchronized population produces a combined coherent signal which is stronger than the individual signals of the unsynchronized population [20]. More tightly synchronized groups of neurons thus exert a stronger phase shifting effect on less tightly synchronized groups of neurons. In previous studies, it has been shown that the ventral and dorsal part of the SCN can desynchronize, in response to short light-dark (LD) cycles, or in response to advanced or delayed light-dark cycles [12], [13], [18], [22]. While the ventral and dorsal SCN remain desynchronized for long periods of time in short LD cycles [22], the dorsal and ventral part resynchronize following a shift of the light-dark cycle. Previous studies have shown that the ventral part of the SCN is dominant in setting the final phase of the SCN, while vice versa, the dorsal part has less effect on the ventral part [12], [13], [18]. In the present paper we show that this ‘dominant’ group is in fact rather small and consider that the high degree of synchrony of this shifted (small) group of neurons may add to it's ability to pull the phase of the larger group of unshifted neurons over to the new phase.

Previous studies have shown that 27% of the SCN neurons in the rat are directly influenced by retinal illumination [23], [24]. The retinohypothalamic tract (RHT) is the major photic input pathway to the SCN [25] and projects predominantly to cells in the ventrolateral SCN [26]–[28] with glutamatergic and PACAP-containing fibers [25], [29]. The ventral (or core) cells of the SCN contain vasoactive intestinal peptide (VIP), gastrin-releasing peptide (GRP) and substance P (SP), which are colocalized to certain degrees [30]–[34]. Not all ventral neurons receive retinal input [35] and it is unknown how photic information is spread from the photorecipient neurons to other regions of the SCN. The percentage of SCN neurons that is directly responsive to retinal illumination with a change in discharge rate corresponds closely with the percentage of neurons that shows the immediate shift in response to the exposure of the delayed light cycle, and it would be interesting to investigate whether these are the same neurons.

Phase advances of the circadian system are known to be more complex, and require more time, than phase delays. Results from our phase advancing studies are consistent with the view that a small subset of neurons shift immediately to the new ZT 6, but also revealed a large group of subpopulations with a broad distribution. The multiunit patterns after the advancing shift showed also substantial variations between the recordings, which is a surprising observation and interferes with an attempt to provide a general quantitative model for phase advances. The reason for the variability between the recorded patterns is unknown. Very few patterns showed two components, other patterns showed one very broad peak, and some patterns showed one peak that was not broader than in control conditions. Also in response to 6 hour phase advances, bimodal peaks were rarely observed [19], suggesting a lack in phase organization also following 6 hour advances. The significant broadening of the mean peak width observed in this study supports the view that advances are accompanied by substantial desynchronization. The desynchronization may contribute to the inertia that is often observed after exposure to an advancing light cycle. The difficulty to adjust to advances has been linked to the input from afferent pathways from extra-SCN areas [15], [19], and it is possible that activation of these input pathways contributes to the phase desynchrony within the SCN.

Previous studies have shown that the dorsal and ventral region of the SCN shift at a different pace in response to a shift of the light-dark cycle [11]–[13], [18]. In molecular studies, various clock genes have been assessed following a phase delay or advance of the light-dark cycle. For instance, the expression of Per1 showed a rapid phase shift immediately after a shifted cycle, whereas the clock gene Cry1 required more time to shift [11], [13], [14], [19]. However, also for rapidly shifting genes, the response showed spatial heterogeneity and the ventral part of the SCN shifted faster than the dorsal part [13]. A more detailed regional analysis revealed that also within the ventral and dorsal regions, differences in phase shifting capacity of Per1 exist [18]. This is consistent with our hypothesis that within the ventral SCN, only a subpopulation of neurons shows immediate shifts in electrical activity.

In conclusion, we propose that phase shifts are brought about by an initial rapid shift of a relatively small subpopulation of neurons which is located in the ventral SCN. The small group of rapidly shifting neurons becomes highly synchronized after the shift. The high degree of synchronization of the shifted neurons may contribute to the dominance of this group in setting the final phase of the pacemaker. Although this mechanism is also visible in our advance studies, the high degree of desynchrony within the SCN after an advance is more striking. The relative difficulty of the circadian system to respond to an advance of the light cycle, compared to a delay, may be the result of the higher degree of desynchronization after an advancing phase shift.

## Materials and Methods

### Ethics Statement

All experiments were performed in accordance to animal welfare law and with the approval of the Animal Experiments Ethical Committee of the Leiden University Medical Center with DEC nr 4085.

### Electrical Activity Recordings in SCN Slices

Male wildtype Wistar rats (Harlan, Horst, The Netherlands) were individually housed in cages that were equipped with a running wheel and entrained to a 12:12 light-dark cycle. Food and water were available ad libitum. When the animals were properly entrained (for at least 3 weeks), the light-dark schedule was delayed by 6 hours by postponing the time of lights-off, or advanced by 9 hours by advancing the time of lights-on [12]. After experiencing a full cycle of the new light regime, animals were decapitated and brains quickly removed. Subsequently, coronal hypothalamic slices (∼400 µm thickness) containing the SCN were prepared and transferred to a recording chamber within 6 min after decapitation, as described previously [36]. Slices were constantly perfused with oxygenated artificial cerebrospinal fluid (ACSF) and maintained at a temperature of 35°C. The submerged slice was stabilized with a resin coated tungsten fork and settled in the recording chamber for ∼ 1 h before electrode placement.

Recording electrodes were placed in the ventral and dorsal SCN in order to obtain multiunit discharge activity patterns from both SCN regions simultaneously. Action potentials were recorded with resin coated metal electrodes (90% platinum 10% iridium, ø75 µm), amplified 10k times and bandpass filtered (300 Hz low, 3 kHz high). The action potentials crossing a preset threshold well above noise (∼5 µV) were counted electronically in 10 s bins by a computer running custom made software. Time of occurrence and amplitudes of action potentials were digitized and recorded by a data acquisition system (Power1401, Spike2 software, CED, Cambridge, UK) and stored for off-line analysis.

### Analysis of Electrical Activity Data

Multiunit electrical activity recordings on day 1 after a shift of the light-dark cycle were used for the quantitative analysis, because on day 1 we can analyze the direct effects of the perturbation on the phase of the SCN subpopulations. Furthermore, the percentage of bimodal peaks was largest on day 1 and a reorganization of the neuronal subpopulations towards the final configuration may already have started on day 3.

Following a delay of the light-dark cycle the electrical activity data exhibiting bimodal activity patterns were used for the quantitative analysis. The recordings were smoothed using a penalized least squares algorithm [37]. The width of each component was determined at the level of the trough between the two peaks. To allow for comparison between different experiments, we also calculated the relative width for each component in the bimodal electrical activity pattern. The difference in component widths was tested for statistical significance with independent t tests (p<0.001).

### Curve Fitting

The area under the curve was determined for each component using manual curve fitting and automatic curve fitting procedures (Igor: http://www.wavemetrics.com and Origin: http://www.originlab.com). In the manual curve fitting method the areas of each component were visually determined in the smoothed bimodal multiunit activity pattern at the level of the trough. For automatic curve fitting, mathematical techniques of the curve fitting algorithms available in Igor (http://www.wavemetrics.com) and in Origin (http://www.originlab.com) were applied to fit the bimodal pattern, and Gaussian functions were fitted to the smoothed curve. The levels of the trough in the multiunit activity pattern functioned as a baseline level for the fitting procedure. When the trough levels on consecutive cycles were unequal, the baseline level was adjusted and was estimated by either a linear function (*f(x)  =  a + bx*) or a cubic function (*f(x)  =  a + bx + cx^2^ + dx^3^*). The fits produced by the linear and cubic function were sufficient, and better than results from a quadratic function. The resulting Gaussian functions that best described the bimodal pattern in multiunit activity were selected, using the lowest chi-square test statistic and the lowest Akaike Information Criterion (AIC) value. The Gaussian function describing the component was characterized as follows
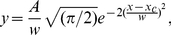
(1)where *A* represents the area under one Gaussian, *w* characterizes the width of the Gaussian function, and *x_c_* represents the time of the peak of the Gaussian function.

For both manual and mathematical fitting, the area for the selected components was subsequently determined relative to the total area under the curve. The relative area for each component was used as an indication for the relative number of action potentials contributing to each component.

### Simulations

Simulations were performed in the Matlab programming environment (The Mathworks: http://www.mathworks.com) using a model that was described previously [38]. In short, extracellular recorded single unit activity patterns were used for the simulations. The peaks of all normalized single unit activity patterns were aligned to determine the mean activity profile of a single SCN neuron [39]. These units were distributed over the circadian cycle according to a Gaussian phase distribution, which is at present the most realistic distribution [40], [41]. Multiunit activity patterns were generated by adding up the equally weighted activity of all single units.

In the current simulations, two components were simulated at the average ZT times of the unshifted and shifted component (ZT 9 and ZT 13, ZT before the shift of the environmental cycle). In these simulations the activity of neurons in both components was equally weighted, because there is no indication that the activity level of SCN units depend on the phase of the cycle [39]–[41]. The multiunit activity pattern was derived from the activity patterns of the two components. Each component was composed as an ensemble of neurons, by distributing a number of single unit activity patterns according to the following Gaussian distribution: 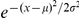. The number of neurons and the width of the distribution could be adjusted for each component separately. The sigma (σ) of a distribution is a measure for the width of a Gaussian distribution, a high value of sigma reflecting a broad distribution and a low value of sigma indicating a narrow distribution.

### Subpopulation Analysis

Activity of neuronal subpopulations in the SCN was analyzed using Matlab (The Mathworks). Subpopulations in the multiunit recordings were selected on the basis of spike amplitude. The amplitude data were divided into 50 equally sized Voltage-amplitude bins reaching from a low spike threshold level, representing a large number of neurons, to the highest threshold including only a few units [40]. As the amplitude of extracellularly recorded spikes is a function of the distance to the electrode tip, the high threshold subpopulation represents a small group of neurons that are located close to each other and close to the electrode tip. Population and subpopulation activity were smoothed and the peak times of the different subpopulations were determined. In the delay studies, these peak times were also determined relative to the time of the trough between both peaks in the bimodal pattern of the multiunit activity recording. All recordings were aligned to the time of the troughs in order to determine the peak times relative to the trough.
